# Zebrafish *foxP2* Zinc Finger Nuclease Mutant Has Normal Axon Pathfinding

**DOI:** 10.1371/journal.pone.0043968

**Published:** 2012-08-24

**Authors:** Lingyan Xing, Kazuyuki Hoshijima, David J. Grunwald, Esther Fujimoto, Tyler S. Quist, Jacob Sneddon, Chi-Bin Chien, Tamara J. Stevenson, Joshua L. Bonkowsky

**Affiliations:** 1 Division of Pediatric Neurology, Department of Pediatrics, University of Utah School of Medicine, Salt Lake City, Utah, United States of America; 2 Department of Neurobiology and Anatomy, University of Utah School of Medicine, Salt Lake City, Utah, United States of America; 3 Interdepartmental Program in Neurosciences, University of Utah School of Medicine, Salt Lake City, Utah, United States of America; 4 Department of Neurology, University of Utah School of Medicine, Salt Lake City, Utah, United States of America; 5 Department of Human Genetics, University of Utah School of Medicine, Salt Lake City, Utah, United States of America; University of Sheffield, United Kingdom

## Abstract

*foxP2*, a forkhead-domain transcription factor, is critical for speech and language development in humans, but its role in the establishment of CNS connectivity is unclear. While *in vitro* studies have identified axon guidance molecules as targets of *foxP2* regulation, and cell culture assays suggest a role for *foxP2* in neurite outgrowth, *in vivo* studies have been lacking regarding a role for *foxP2* in axon pathfinding. We used a modified zinc finger nuclease methodology to generate mutations in the zebrafish *foxP2* gene. Using PCR-based high resolution melt curve analysis (HRMA) of G0 founder animals, we screened and identified three mutants carrying nonsense mutations in the 2^nd^ coding exon: a 17 base-pair (bp) deletion, an 8bp deletion, and a 4bp insertion. Sequence analysis of cDNA confirmed that these were frameshift mutations with predicted early protein truncations. Homozygous mutant fish were viable and fertile, with unchanged body morphology, and no apparent differences in CNS apoptosis, proliferation, or patterning at embryonic stages. There was a reduction in expression of the known *foxP2* target gene *cntnap2* that was rescued by injection of wild-type *foxP2* transcript. When we examined axon pathfinding using a pan-axonal marker or transgenic lines, including a *foxP2*-neuron-specific enhancer, we did not observe any axon guidance errors. Our findings suggest that *foxP2* is not necessary for axon pathfinding during development.

## Introduction

Language impairment is central to autism and the autistic spectrum disorders, and is a major component in many neurodevelopmental disorders including Angelman syndrome and Fragile X syndrome [1]. Unraveling the genetic pathways and neural circuitry involved in language development is important for understanding these different disorders. However, only one gene, *FOXP2*, has been linked specifically with the normal development of language [2]. This gene, a forkhead-box transcription factor, was originally identified in a family with a severe speech and language disorder, and has subsequently been identified in other patients as well [2]–[8]. Haploinsufficiency for *FOXP2* in humans leads to defects in grammatical language construction, as well as in the sequencing of orofacial movements required for speech articulation [9]. Studies on the human *FOXP2* mutant pedigrees, using functional and volumetric magnetic resonance imaging (MRI), show abnormalities of the basal ganglia, cerebellum, and prefrontal cortex [10]–[12].

However, it is not clear what the primary role of *FOXP2* in the central nervous system (CNS) is, nor how that leads to impaired language development. Further, both pre- and post-natal functions for *FOXP2* have been proposed. In mice, *Foxp2* heterozygotes have impaired motor learning [13], while homozygotes have a smaller cerebellum [13] and a disorganized Purkinje cell layer in the cerebellum [14], suggesting a developmental role for *Foxp2*. In contrast, knockdown of *FoxP2* with lentivirus-mediated RNA interference (RNAi) in Area X of songbirds leads to inaccurate vocalizations [15], consistent with a post-natal role.

Additional support for a developmental function of *FOXP2* has come from studies implicating a role for *FOXP2* in axon pathfinding. First, *in vitro* chromatin immunoprecipitation (ChIP) showed that *CNTNAP2*, a member of the neurexin superfamily, was a target of *FOXP2* [16]. *CNTNAP2* is associated with specific language impairment as well as with autism [17]–[19]. *CNTNAP2*, as well as other autism-associated genes, show defects in the normal development of connectivity [19]–[21]. Second, ChIP analysis showed that *FOXP2* regulated genes involved in axon guidance, including *EPHA2* and *SEMA3B* [22], [23]. Third, cell culture studies demonstrated that normal function of *Foxp2* was necessary for neurite outgrowth [24].

We decided to address whether *FOXP2* has a role in regulating axon guidance *in vivo*. We used a zebrafish model because of its rapid CNS development and relative ease for imaging and analysis of axon pathfinding. Zebrafish foxP2 has 86% protein similarity to human FOXP2 [25], and its CNS expression pattern is conserved in the telencephalon, basal ganglia, and cerebellum to that of humans, mice, songbirds, and frogs [25]–[30]. Previously we had tried to knock down *foxP2* expression using morpholinos in zebrafish embryos. However, five different morpholinos had embryonic toxicity, leading to early lethality (JLB, unpublished data) that was not rescued using a morpholino against *p53* [31].

In this paper we generated and screened zinc finger nucleases (ZFNs) against zebrafish *foxP2* using a modified bacterial 1-hybrid screen. Mosaic G0 injected fish were identified using high resolution melt analysis (HRMA) PCR of somatic DNA (fin-clip), and we describe our use of HRMA PCR for screening and identification of mutants. We generated three frameshift *foxP2* mutant alleles: an 8 bp deletion, a 17 bp deletion, and a 4 bp insertion. The three mutant alleles were homozygous mutant and fertile, and characterization of CNS development revealed no changes in apoptosis, proliferation, patterning, or specification. To analyze pathfinding we used both pan-axonal immunohistochemistry, as well as neuron-type specific transgenic reporter lines. We found that disruption of *foxP2* in zebrafish did not affect axon pathfinding during development. Our results demonstrate the importance of *in vivo* validation of ChIP and *in vitro* studies, and are concordant with other studies suggesting a role for *foxP2* in synapse development [13], [32].

## Results

### Zinc Finger Nuclease (ZFN) Generation, Injection, and Screening

**Figure 1:**
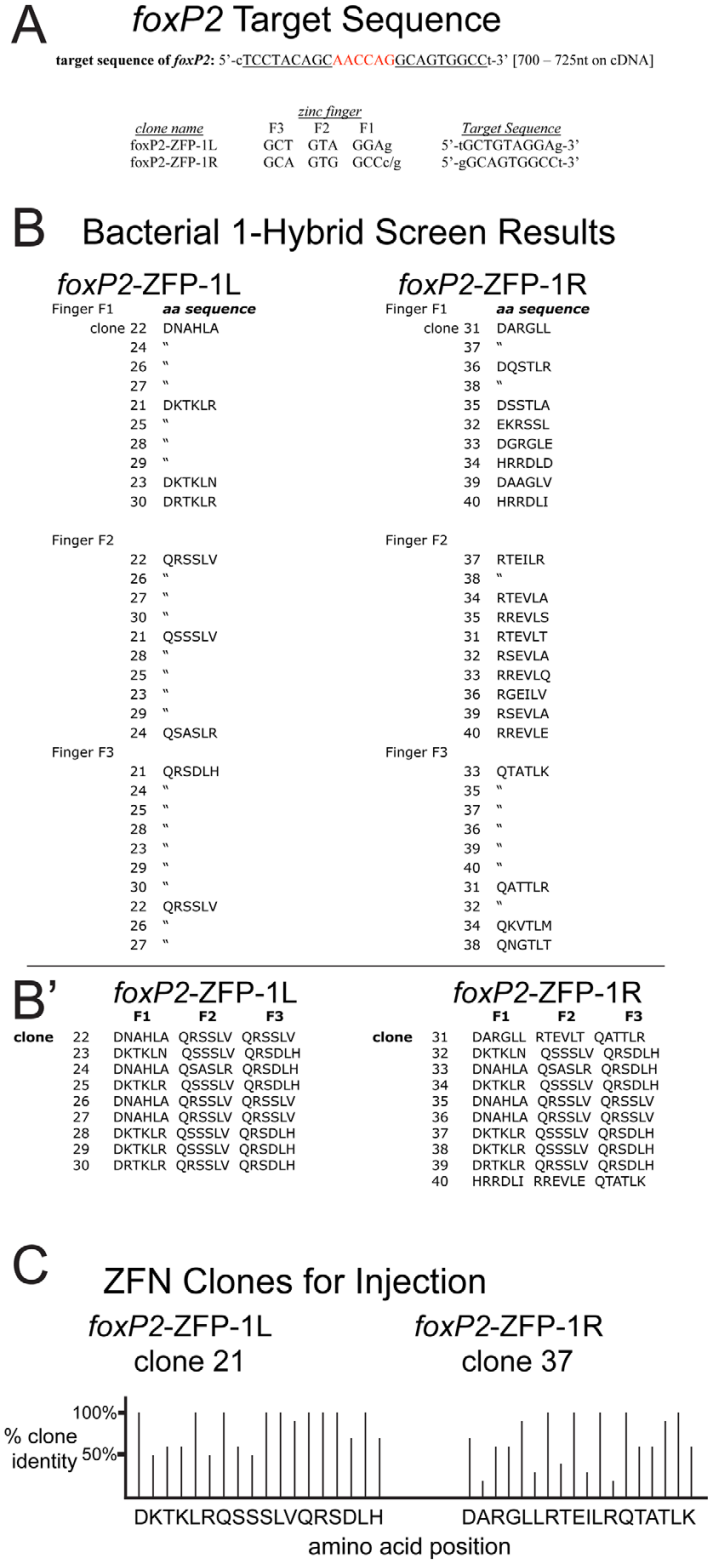
Targeting and selection of ZFNs. (A) *foxP2* cDNA sequence, nt 700–725, with target cleavage region in red, and ZFN binding targets underlined. Codons for design of the left and right zinc finger proteins (ZFP) are shown below with their respective target sequence. (B and B′) Amino acid sequences of the 10 clones from bacterial 1-hybrid selection, arranged by clone number, zinc finger, and left or right; or (B′) selected clones shown sequentially. (C) Graphical representation of the relative amino acid frequency compared to other selected ZFN clones, at each position, in the two ZFN clones chosen for injection.

We designed ZFNs against a region in exon 2 of the zebrafish *foxP2* cDNA using the target prediction program ZiFiT (http://bindr.gdcb.iastate.edu/ZiFiT) (Figure 1A). The site in exon 2 was the only ZFN target 5′ of the functional domains including the forkhead domain and zinc finger domain, as the other acceptable ZFN sites were 3′ to these domains. OPEN pool PCR amplification, generation of the three-finger zinc finger protein libraries, reporter plasmid preparation, and bacterial 1-hybrid screening was performed as described [33]. We screened bacterial 1-hybrid libraries with titers of 2.8×10^7^ and 1.1×10^7^ cells/plate, for the right and left fingers, respectively. We picked and sequenced the selected zinc finger proteins for 10 clones for each ZFN clone (20 clones total) and compared the amino acid sequences (Figure 1B, B'). The recovered clones from the library screening were selected from plates with middle to high stringency: the concentration of carbenicillin was 100 μg/mL and the concentration of 3-AT ranged from 20–30 mM. For our final choice of clones for the left and right ZFNs, we chose the clones that had the highest percentage of amino acids in common with the other clones at the specific positions in the zinc finger (Figure 1C). We hypothesized that this conservation was indicative of a relative selection for this amino acid at a particular position. For the “left” ZFN clone (clone #21), every amino acid was found in at least 50% of the selected clones, and in half of the positions every clone shared 100% identity. The right ZFN clone (clone #37) had less conservation, but still 1/3^rd^ of the sites had perfect conservation.

**Figure 2:**
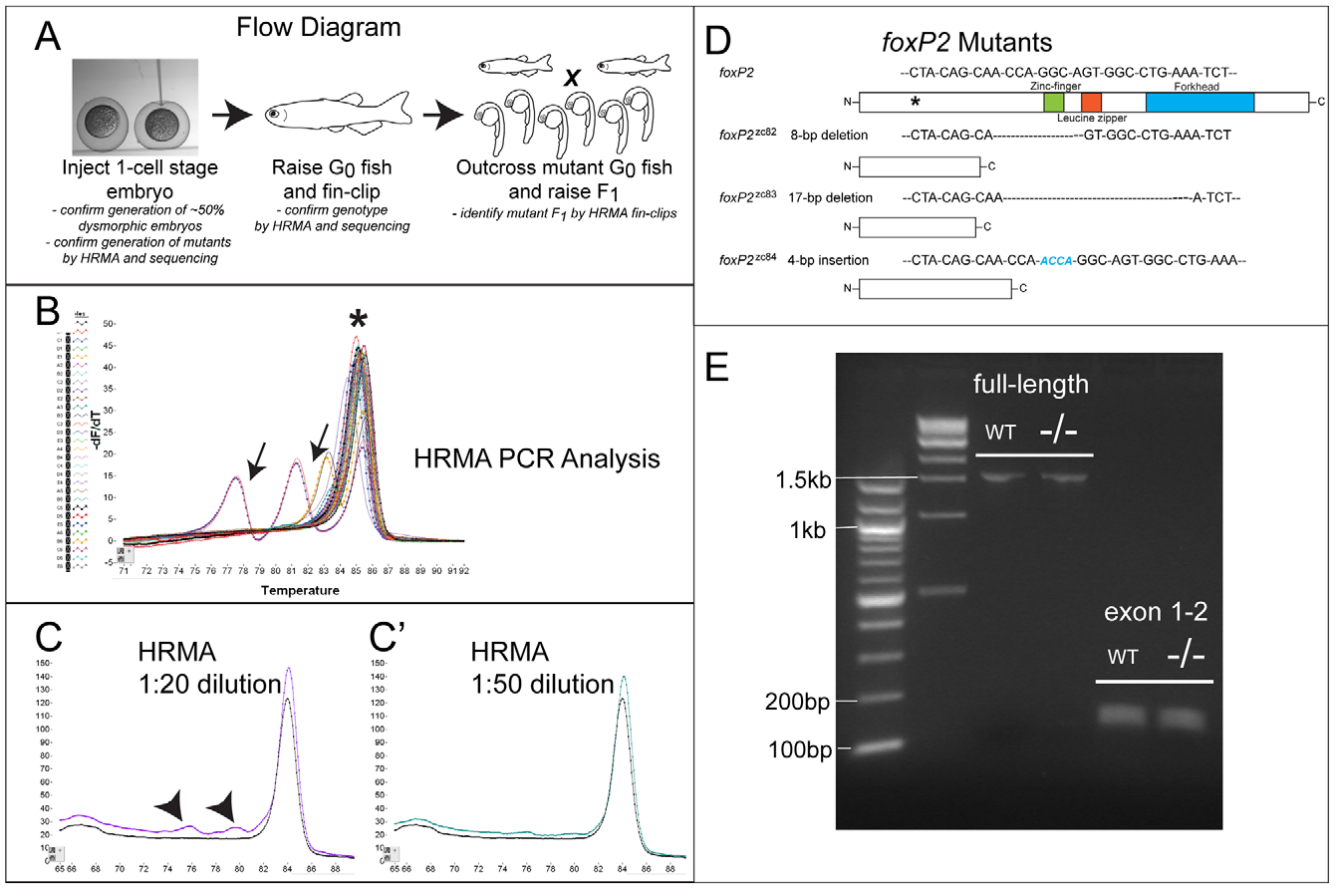
Flow diagram for generation of ZFN mutants, and HRMA analysis. (A) Flow diagram illustrating steps in generation and identification of fish carrying ZFN-induced mutation. (B) Illustrative melt-curve (“HRMA” high-resolution melt analysis) of F1 generation *foxP2* ZFN fish. Y-axis corresponds to the differential of the change (decrease) in fluorescence; X-axis is the temperature (°C). Each colored line is a different sample, arrows point to samples with mutations in the *foxP2* amplicon; asterisk is the wild-type product. There is some variability in the melt temperature of the wild-type amplicon because of minor variations in starting template amount and salt concentrations. (C-C′) Melt-curves of dilutions of mutant *foxP2* DNA. Abnormal melt curves (arrowheads) are detected at dilutions of 1∶20 mutant:wild-type DNA, but not (or very minimally) at a dilution of 1∶50. (D) Schematic diagram of foxP2 protein domains; asterisk shows the targeted region for ZFN mutagenesis; sequence above the picture is the nucleotide sequence targeted. Below are shown the three alleles, with the sequence of the mutation, and a picture of the predicted protein. (E) RT-PCR from wt or *foxP2* homozygous mutant embryos at 72hpf, with primers for the full-length transcript, or encompassing exons 1–2. No alternative splice variants were noted, and sequencing of the *foxP2* mutant PCR product showed that the mutations led to the predicted out-of-frame sequence.

Clones #37 and #21 were subcloned into the pCS2-FokI-DD and -RR FokI nuclease expression vectors (pCS2-Flag-TTGZFP-FokI-DD and pCS2-HA-GAAZFP-FokI-RR [34]), and injected as mRNA into zebrafish embryos (Figure 2A). Initially we injected 20, 80, and 250 pg of each ZFN into 1-cell stage embryos, and at 48 hours post-fertilization (hpf) assessed the percentage of embryos that showed a dysmorphic or “monster” appearance [34]. We found that injections at 80pg each resulted in approximately 50% dysmorphic embryos, and we used this amount for subsequent experiments. We confirmed by HRMA PCR (discussed below) performed on pools of 4–10 embryos each (Figure 2B), and by subsequent selection of individual clones for sequencing, that we were inducing mutations in the *foxP2* target locus. We raised 25 G0 injected embryos to adulthood, and screened by HRMA PCR on fin-clip DNA samples. We identified four G0 fish with abnormal melt-curves in somatic DNA; these fish were crossed to wild-type fish, and pools of 4 embryos each were screened again by HRMA PCR. Two of the identified G0 fish did not yield any mutant offspring, while two fish (of the four) produced mutant offspring, and were crossed to subsequently give rise to the three different *foxP2* alleles. For the two G0 fish which did not yield mutant offspring, the mutation may not have been present in the germline, it may have been present at very low rates, or the HRMA PCR result may have been falsely abnormal. In the F1 offspring of the two mutant G0 parents, the relative frequency of mutant offspring was 7% and 25%.

HRMA PCR was performed by designing a small amplicon centered on the *foxP2* ZFN target locus, and performing PCR and melt-curve analysis of the PCR product [35]. Following amplification in the presence of a fluorescent dye that binds double-stranded DNA, a “melt” of the PCR product either yields a single peak (Figure 2B, asterisk) corresponding to a single wild-type product, or to multiple peaks and/or a broader, shifted peak corresponding to heteroduplexes of wild-type and mutant PCR product (Figure 2B, arrows), which melt at a lower temperature [36]. By performing a dilution analysis of mutant DNA into wild-type DNA, we determined that we could reliably detect mutant samples when mutant DNA was present in a proportion of 1∶20, but not to 1∶50 (Figure 2C-C′, arrowheads). Thus, HRMA PCR will be able to detect founder G0 fish if mutant cells are present in more than 1/20–50^th^ of the total cells. Similarly, screening F1 embryos should be limited to no more than 15–20 embryos concurrently (20 embryos = 40 copies of a genomic locus).

### *foxP2* Mutant Alleles

We identified three mutant alleles: *zc82* (8bp deletion), *zc83* (17bp deletion), and *zc84* (4bp insertion), which lead to out-of-frame proteins with early stop codons at amino acids 137, 134, or 164 (out of 697 amino acids) for each mutant (Figure 2D). The three different alleles each generate a different amino acid sequence downstream of the mutation prior to the stop, as the alleles each have a slightly different location of where the deletion or insertion has occurred. We outcrossed mutant alleles for successive generations to reduce the potential problem of off-target mutations [37]. We assessed both *in trans* heterozygous (heteroallelic) and *in cis* homozygous mutants, but for the experiments shown here we used heteroallelic mutants in order to avoid possible off-target background effects. We performed experiments by crossing heteroallelic parents to one another, and genotyping their embryos with HRMA. The use of *foxP2* heteroallelic mutant parents prevented possible rescue or amelioration of effects due to maternal contribution of wild-type transcript from a heterozygous parent.

To demonstrate that the mutations led to disruption of the normal transcript we performed RT-PCR followed by sequencing of the products. We found a single transcript was generated in mutant embryos which included the mutated exon 2 (Figure 2E). We cloned and sequenced the PCR product from exon 1–2 amplification, and found that the mutation led to the predicted shift in codon reading frame. These results suggest that the ZFN-induced *foxP2* mutants will lead to expression of truncated, mutant protein products lacking the zinc-finger, leucine zipper, and forkhead domains. Truncation of human *FOXP2* by a premature nonsense mutation prior to these domains has been shown to lead to loss of functional protein [38], [39], suggesting that our mutations are in fact nulls.

**Figure 3:**
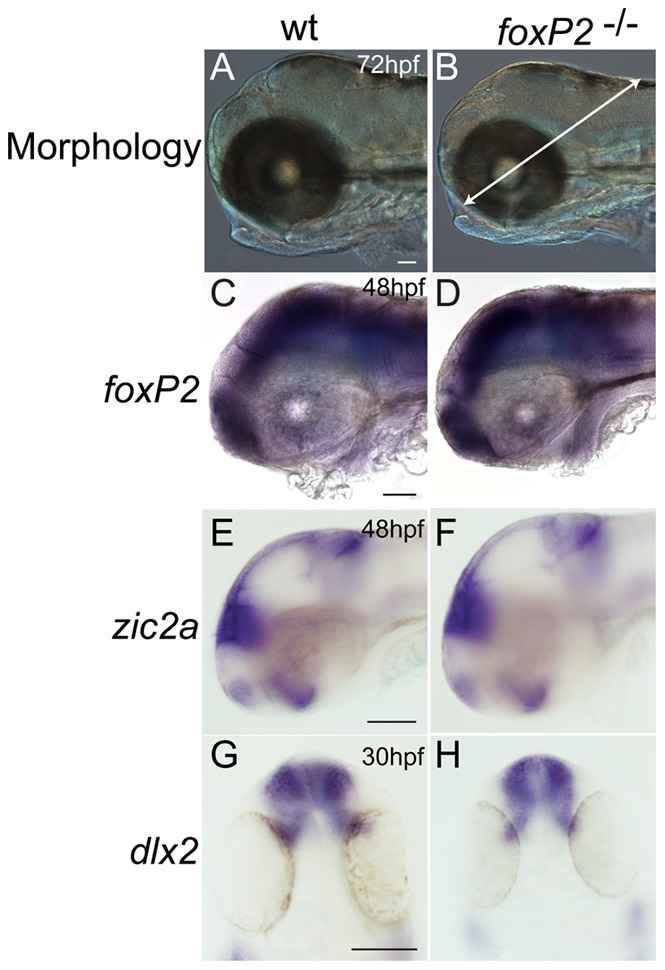
*foxP2* mutant has normal morphology and CNS patterning. Whole-mount embryos, brightfield images, scale bar  = 50 μm. Lateral views, rostral to the left (A-F); dorsal views, rostral to the top (G, H). (A, B) Gross head morphology is unchanged in mutants at 72hpf. Arrow in (B) shows line used to measure brain size, from the midbrain-hindbrain boundary to the edge of the head by dissecting the midpoint of the lens. (C-D) mRNA expression of *foxP2* transcript is unchanged in intensity and pattern in *foxP2* mutants. (E-H) *in situ* expression patterns and intensity of *zic2a* and *dlx2* is unchanged in mutants compared to wild-type embryos.

**Figure 4:**
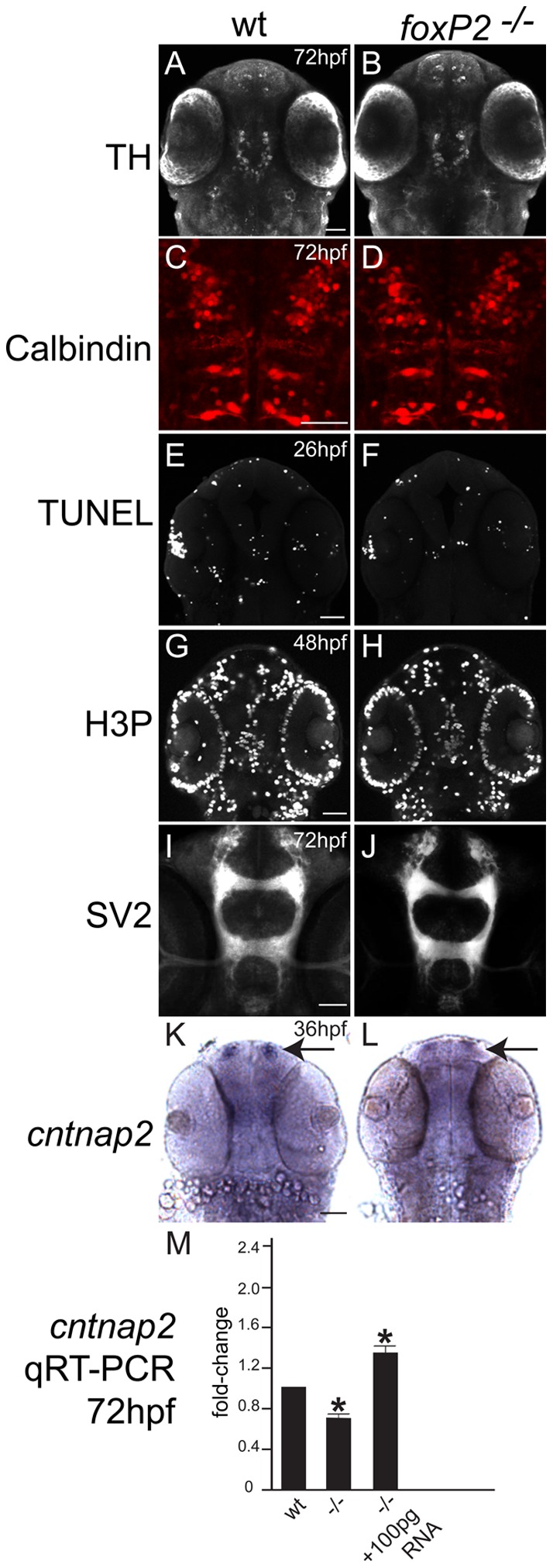
*foxP2* does not affect neuron specification, apoptosis, or proliferation, but does affect *cntnap2* expression levels. Confocal z-stack images, rostral to the top, scale bars 50 μm. (A, B, E-H, K-L) ventral views. (C, D) dorsal views. (*A*, *B*) TH immunohistochemistry at 72hpf shows no difference in WT and mutants in diencephalic dopaminergic neuron pattern or number. (C, D) Calbindin immunohistochemistry in the dorsal hindbrain at 72hpf shows similar patterns and numbers of Purkinje neurons in WT and mutants. (E, F) TUNEL staining for apoptotic cells at 26hpf in the brain shows no difference in pattern or number of cells between WT and mutants. (G, H) Detection of proliferation by H3P staining is similar in WT and mutants at 48hpf. (I, J) Neuropil distribution and intensity visualized with anti-SV2 synaptic vesicle protein antibody at 72hpf is similar in WT and mutants. (K, L) *cntnap2 in situ* expression pattern at 36hpf shows less expression in mutant embryos, more noticeably in the telencephalon (arrows). (M) quantitative RT-PCR at 72hpf confirms decreased expression of *cntnap2* in mutants, which is rescued by injection with full-length *foxP2* mRNA (* *p*<0.05). Y-axis indicates fold-change.

Homozygous mutant fish were viable and fertile, including both heteroallelic (*foxP2* ^zc82/zc83^, *foxP2* ^zc82/zc84^, *foxP2* ^zc83/zc84^) and homozygous (*foxP2* ^zc82/zc82^, *foxP2* ^zc83/zc83^, *foxP2* ^zc84/zc84^) alleles. There were no obvious changes in overall body morphology of *foxP2* mutants. By *in situ* analysis, there was no change in *foxP2* expression pattern or overall intensity of expression in mutants (Figure 3 C, D), which is not unexpected since we expect normal production of mRNA transcript based on the nature of the alleles. We also measured brain size at 72hpf, and found that mutants had no difference compared to their wild-type siblings (325 vs. 323 μm, SEM 3.5 and 2.6, *p* = 0.64) (Figure 3A, B). Further, there were no changes in *foxP2* mutants in the CNS of apoptosis or of proliferation, as assayed using TUNEL (terminal deoxynucleotidyl transferase dUTP nick-end labeling) and anti-H3 phosphohistidine (H3P) antibody at 48hpf and 72hpf, respectively (Figure 4E-H).

### CNS development and patterning

To broadly assess CNS patterning we performed *in situs* for *dlx2* and *zic2a* at 30hpf and 48hpf, respectively. *dlx2* and *zic2a* are widely expressed and regulate development of many cell types in the CNS [40]–[42]. No changes in expression patterns of *dlx2* and *zic2a* were detected in *foxP2* mutants compared to wild-type (Figure 3E-H). This suggests that *foxP2* does not have a role in overall CNS patterning.

We examined neuron specification in detail for Purkinje cells and dopaminergic neurons, because of a proposed role for *FoxP2* in their development and function [10]–[14]. Using immunohistochemistry for calbindin to label Purkinje cells of the hindbrain, and for tyrosine hydroxylase to label dopaminergic neurons of the diencephalon, we found no apparent differences in cell morphology, number, or organization in *foxP2* mutants (Figure 4 A–D). Therefore, in zebrafish *foxP2* does not appear to have a role in development of cerebellar or dopaminergic neurons.

*Foxp2* has also been proposed to regulate synapse formation [13], [32]. We used immunohistochemistry for the pre-synaptic vesicle protein SV2 [43], and found no difference in neuropil development in *foxP2* mutants (Figure 4 I, J). However, *CNTNAP2*, a neurexin-family member identified as an *in vitro* target of *FOXP2* with a potential role in neuronal migration and development of CNS connectivity [16], [19], had decreased expression at 36hpf (Figure 4K, L, arrows). This decrease was developmentally persistent and was confirmed by quantitative RT-PCR to be down-regulated to 72% of normal levels in 72hpf *foxP2* mutants (standard error of the mean 0.078; *p*<0.05) (Figure 4M). To confirm the specificity of this effect we injected *foxP2* transcript into mutant embryos at the 1-cell stage transcribed from a full-length cDNA clone [25]. At 72hpf RNA was prepared from embryos (wild-type, mutant, or mutant injected with mRNA) that were morphologically indistinguishable, and a resultant rescue of *cntnap2* levels was found (Figure 4M).

For both the *in situs* and the RT-PCR of *cntnap2* we used heteroallelic (trans-heterozygous) parents to generate the mutant off-spring (zc82/zc84×zc82/zc84). This was designed to: i. prevent any maternal mRNA contribution from obscuring results; and ii. to prevent any non-specific background effects of off-target mutations caused by the zinc-finger nucleases. The wild-types were generated by crosses between heteroallelic heterozygous parents (i.e. zc82/+× zc84/+), and confirmed by HRMA PCR. Thus although they were not “litter” (clutch) mates, they were of the same genetic background. Thus, our zebrafish *foxP2* mutant does recapitulate some aspects of CNS dysregulation noted in other systems.

### Axon pathfinding

**Figure 5:**
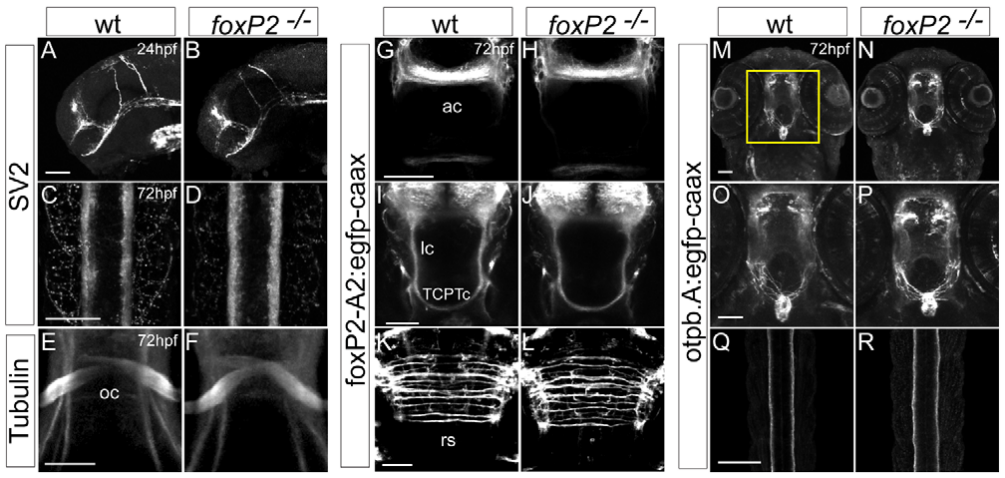
*foxP2* does not affect axon pathfinding. Confocal z-stack images of whole-mount embryos, scale bars 50 μm, show no difference between wild-type and *foxP2* mutant (−/−) embryos for axon pathfinding using a variety of axonal labels. (A–D) anti-SV2 immunohistochemistry at 24hpf, lateral views of the brain, rostral to the left (A, B) and 72hpf, dorsal views of the spinal cord, rostral to the top (C, D). (E, F) anti-acetylated tubulin immunohistochemistry at 72hpf, ventral views of the optic chiasm. (G-L) GFP immunohistochemistry at 72hpf in Tg(*foxP2-enhancerA.2:egfp-caax*) embryos that labels *foxP2* neurons show no pathfinding errors in anterior commissure (ac), longitudinal commissures (lc), tract of the commissure of the posterior tuberculum (TCPTc), or reticulospinal axons (rs). (M-R) GFP immunohistochemistry at 72hpf in Tg(*otpb.A:egfp-caax*) embryos for visualization of dopaminergic and neuroendocrine projections (M, N, with insets shown in O, P) in the brain, and dopaminergic axon tracts in spinal cord (Q, R).

An outstanding question for *foxP2* is whether it regulates the development of CNS connectivity. To address this, we analyzed whether *foxP2* is required for normal axon pathfinding *in vivo*. We used both acetylated tubulin antibody and anti-SV2 to label all axon tracts, as well as enhancers that specifically express membrane-targeted GFP in subsets of neurons, including *foxP2* neurons (*foxP2-enhancerA.2:egfp-caax*) [44] and axons of diencephalic dopaminergic neurons (*otpb.A:egfp-caax*) [45], [46]. We analyzed axon tracts at 24hpf and 72hpf, including the anterior and post-optic commissures, optic chiasm, tract of the commissure of the posterior tuberculum (TCPTc) [47], dopamine neuron projections, projections to the pituitary, reticulospinal axons, and spinal cord projections. We did not observe any axon pathfinding errors in *foxP2* mutants (Figure 5 A–R). Our findings show that *foxP2* does not play a role in pathfinding during development, assayed using a variety of markers for different axon tracts.

## Discussion

Determining the function of *FOXP2* in the CNS has been difficult, and different groups [13], [15], [24], [28], [32] have proposed a variety of roles, including regulation of synaptic plasticity in striatal neurons, development of Purkinje cells for motor-skill learning, and the control of connectivity development [24]. However, studies have been constrained by the use of primarily *in vitro* data, or by a lack of analysis of early CNS development, including basic patterning. Further, no analysis has been done whether *FOXP2* controls the development of axon pathfinding. Since in humans language ability depends on connections between different language areas [48], we had hypothesized a role for *FOXP2* in axon guidance.

In the work we generated *foxP2* mutants in zebrafish using a modified zinc finger nuclease (ZFN) protocol [33], and recovered 3 mutants alleles from 25 injected G0 fish. The use of ZFN technology in zebrafish is important for analysis of gene function. For example, our own previous analysis of *foxP2* in zebrafish using morpholinos led to conflicting results: 5 of 6 different morpholinos had a severe, early embryonic phenotype with tissue necrosis that began shortly after gastrulation, whereas a 6^th^ morpholino caused no observable phenotype. We demonstrated that in the *foxP2* mutant alleles we only had mutant mRNA transcript generated, with no evidence for alternate splicing. While we can not exclude the possibility that other *foxP2* splice variants might be generated that lack the ZFN-induced mutation of exon 2, at least during this critical time period of axon pathfinding we only obtain a single RT-PCR product that contains exon 2.

We tried a variety of commercially available antibodies generated against mouse and human FoxP2, as well as a polyclonal antibody directed against two different peptide epitopes from the zebrafish foxP2 protein, we were unable to find an antibody that was specific (JLB, LX, data not shown). In particular, with the polyclonal antibodies we raised against zebrafish peptide epitopes, there was no signal above background on whole-mount or cryostat section immunohistochemistry. On western blots, the zebrafish-directed antibodies gave numerous bands that did not correspond in size to the predicted zebrafish foxP2 protein. Following pre-incubation with the peptides used to raise the antibodies, there was no loss of an obvious band that might correspond to foxP2. The lack of demonstration of a protein null is a limitation. However, our indirect evidence based on RT-PCR and sequencing, as well as the down-regulation of *cntnap2* and the ability of full-length *foxP2* transcript to rescue the *cntnap2* phenotype, argue that our alleles are mutants.

In addition, our ability to efficiently detect mutant fish was enhanced by our use of HRMA PCR analysis, including screening the G0 fish by analysis of somatic (fin-clip) DNA. This permitted us to analyze a subset of the G0 fish and limit the number of different animals that we needed to cross and screen. HRMA permits rapid genotyping, and we were able to use the patterns of the different melt-curves to distinguish between the different *foxP2* alleles we generated. Using fin-clip followed by HRMA PCR of G0 fish offers a potential short-cut to detect ZFN founders. However, in our experiments we did not examine the founder rate in G0 fish that were fin-clip PCR negative. Thus, we can not exclude the possibility that our method does not enrich for founders and instead reflects chance.

In contrast to work on *Foxp2* in mouse, we found that the *foxP2* mutant zebrafish were viable and fertile, with no changes in CNS patterning or specification, normal axon pathfinding, and minor changes in synapse development. We did not find any abnormalities in specification of Purkinje or dopamine neurons, or in cerebellar development [13], [14], [32]. We also examined in detail effects of loss of *foxP2* on apoptosis, patterning, and CNS patterning and specification, and found no effects in mutant animals. While we can not exclude that *foxP2* might exert more subtle effects on axon pathfinding that our assays did not detect, we have demonstrated that our transgenic lines can detect relatively minor axon pathfinding errors in other experimental paradigms [49].

What explains the difference in phenotypes from zebrafish to mouse? These results are unlikely to be explained by the presence of a second closely related protein in zebrafish, since genomic sequence data as well as our own RT-PCR results do not show any evidence for a second *foxP2* ortholog (JLB, unpublished). There are two *foxP1* orthologs in zebrafish (JLB, unpublished data; [50]), and as FoxP1 can act cooperatively with FoxP2 [51], [52], it is possible that the *foxP1* orthologs in zebrafish could functionally compensate for loss of *foxP2*. However, it is not clear then why in mouse Foxp1 is unable to compensate for Foxp2 function. Finally, *foxP2*'s role in CNS development may have changed over evolution, with a critical role in mouse viability, for example in lung development [52].

Our two primary findings were first, that *foxP2* mutants did not have changes in axon pathfinding. This is in contrast to the results suggesting a role for *Foxp2* in neurite outgrowth, and in regulation of axon guidance genes [24]. This finding must be tempered in that we can not exclude an axon guidance role for *foxP2* that is compensated by one of the *foxP1* orthologs. Second, we found that in *foxP2* mutants there was a decrease in *cntnap2* expression levels, which is opposite to the cell culture results showing that loss of *FOXP2* led to an increase in *CNTNAP2* expression [16]. Our results suggest caution in interpretation of *in vitro* data, although it is possible that zebrafish *foxP2* has different CNS roles than mouse *Foxp2*. However, given the conservation of expression domains and protein similarity in zebrafish, argue at least in part against a widely divergent CNS function of *foxP2* in zebrafish. Future studies with *foxP2* will need to examine in greater detail its role in synaptic development, given our findings of decreased *cntnap2* expression levels, and data from mice and humans on this function [13], [22], [23].

## Materials and Methods

### Ethics Statement

All zebrafish experiments were performed with supervision and in strict accordance of guidelines from the University of Utah Institutional Animal Care and Use Committee (IACUC), regulated under federal law (the Animal Welfare Act and Public Health Services Regulation Act) by the U.S. Department of Agriculture (USDA) and the Office of Laboratory Animal Welfare at the NIH, and accredited by the Association for Assessment and Accreditation of Laboratory Care International (AAALAC). This study was approved by the University of Utah IACUC (protocol #11–06005).

### Fish stocks and embryo raising

Adult fish were bred according to standard methods. Embryos were raised at 28.5°C in E3 embryo medium and staged by time and morphology [53]. For *in situ* or immunohistochemistry staining, embryos were fixed in 4% paraformaldehyde (PFA) (in PBS) overnight (O/N) at 4°C, washed briefly in PBS, dehydrated stepwise from PBS to 100% MeOH, and stored in 100% MeOH at −20°C until use.

Transgenic fish lines used in this paper were the following: Tg*(otpb.A:egfp-caax)* (official ZFIN nomenclature Tg(otpb:1EGFP)^zc49^) [e3] and Tg(*foxP2-enhancerA.2:egfp-caax*)^zc69^ [44], [49]. *foxP2* mutant alleles are *zc82* (8 bp deletion), *zc83* (17 bp deletion), and *zc84* (4 bp insertion) (Figure 2). Injection of DNA constructs and raising of stable transgenic lines was performed as described [44], [54], [55]. Lines are available upon request. Mutant allele sequences have been deposited in ZFIN.

### *In situ* hybridization and Immunohistochemistry

Whole-mount *in situ* labeling and immunohistochemistry were performed as described [25], [44]. Antibodies and dilutions were rabbit polyclonal anti-tyrosine hydroxylase 1∶400 (Millipore); mouse anti-acetylated tubulin 1∶250; goat anti-mouse Alexa 488 1∶500 (Invitrogen); rabbit anti-calbindin 1∶150 (Swant); rabbit anti-H3P (Millipore), 1∶500; mouse anti-SV2 (Developmental Studies Hybridoma Bank) 1∶300; and Cy-3 anti-rabbit 1∶400. Apoptotic cells were stained with TUNEL (Millipore) as described [56].

### Microscopy and image analysis

Image acquisition and analysis were performed as described previously [44]. Embryos were processed and placed in a solution of 80% glycerol/20% PBST, and mounted on a glass slide with a #0 coverslip. NIH ImageJ software was used to merge slices to create maximal intensity z-stack projections. Brain size was calculated using whole-mount images of embryos at 72hpf; distance was calculated in ImageJ from the midbrain-hindbrain boundary to the edge of the head by a line dissecting the midpoint of the lens (Figure 3B).

### cDNA and quantitative RT-PCR

Total RNA from 72hpf embryos was extracted using Trizol (Invitrogen), purified (Qiagen RNeasy mini-columns), and reverse-transcribed (SuperscriptTM III First-strand Invitrogen kit). Primers for *foxP2* were used to amplify either the entire coding region (forward 5′-AGCAGTGAAGTAAGCGCAGTCGA-3′; reverse: 5′-AGCGGCAAAGTGGTCTCCGC-3′), or the region encompassing exons 1 and 2 (forward primer: 5′-AGCAGTGAAGTAAGCGCAGTCGA-3′; reverse primer: 5′-CGCTGTTTGTCGTTGTTCTTTGG-3′). PCR product of exon 1–2 amplification from *foxP2* homozygous mutants were cloned and sequenced.

RT-qPCR reaction for *cntnap2* was performed on 72hpf embryos on two separate occasions. The reaction mix included SYBR Green, primers, and cDNA templates; conditions were 95°C for 10 min, followed by 38 cycles of 95°C for 30 s, 60°C for 30 s and 72°C for 30 s. Each reaction was performed in triplicate and the mean of replicates was calculated; results were normalized to the mRNA level of *cntnap2* in wild-type embryos with β-actin transcript levels as a control using the Pfaffl method [57]. Primers were forward 5′- CCAGCTGTTTGTAGGTGCTTCGGG-3′ and reverse 5′- CGCACCAGCGTCCCACTCTC-3′.

### DNA preparation, Genomic DNA PCR, HRMA, and Dilution Analysis

Genomic DNA was prepared as described [35]. PCR reactions were performed in 96-well, hard-shell plates (Bio-Rad, Inc.) in 10 μl volume: 4μl master mix including Taq polymerase, dNTPs, magnesium chloride, and fluorescent double-stranded DNA binding dye (LCGreen PLUS) (Lightscanner Master Mix- Idaho Technology, Inc.), 5 pmol each primer (forward primer: 5′-CCTACAAGCAGCAAGGCA-3′; reverse primer: 5′-TGTCGTTGTTCTTTGGAGATT-3′), and genomic DNA template extracted from DNA lysis buffer [35]. PCR reactions were covered with 30 μl mineral oil. PCR cycling conditions were: 2 min at 95°C, followed by 28 cycles of 10s at 95°C, 25 s at 61°C, ending with 95°C for 30 s and cooled to 15°C. HRMA was performed on a LightScanner-96 instrument (Idaho Technology, Inc.), from 60°C to 97°C with a temperature transition rate of 0.1°C/s. The melt curves were normalized using temperature ranges of 74–75°C and 87–88°C. Genotypes can be distinguished on the basis of their respective melt temperatures [58]; following initial confirmation by DNA sequencing.

Dilution analyses were performed in triplicate. Genomic DNA was prepared in the standard fashion; DNA concentrations were determined, and corresponding amounts of wild-type and *foxP2* mutant DNA were then mixed. Dilutions of 1∶2, 1∶5, 1∶10, 1∶20, 1∶50, and 1∶100, mutant:wild-type DNA sample were prepared, and HRMA PCR was then performed.

### OPEN selection of zinc finger arrays and bacterial 1-hybrid selection

We designed ZFNs against a region in exon 2 of the zebrafish *foxP2* cDNA using the target prediction program ZiFiT (http://bindr.gdcb.iastate.edu/ZiFiT) (Figure 1A). OPEN pool PCR amplification, generation of the three-finger zinc finger protein libraries, reporter plasmid preparation, and bacterial 1-hybrid screening was performed as described [33]. Final ZFN clones were prepared in pCS2-FokI-DD and pCS2-FokI-RR nuclease expression vectors (pCS2-Flag-TTGZFP-FokI-DD and pCS2-HA-GAAZFP-FokI-RR were from Addgene, plasmids #18755 and 18754); transcribed mRNA was injected into 1-cell stage zebrafish embryos at 80pg each.
